# Traffic-related air pollution and respiratory symptoms among asthmatic children, resident in Mexico City: the EVA cohort study

**DOI:** 10.1186/1465-9921-9-74

**Published:** 2008-11-16

**Authors:** Maria-Consuelo Escamilla-Nuñez, Albino Barraza-Villarreal, Leticia Hernandez-Cadena, Hortensia Moreno-Macias, Matiana Ramirez-Aguilar, Juan-Jose Sienra-Monge, Marlene Cortez-Lugo, Jose-Luis Texcalac, Blanca del Rio-Navarro, Isabelle Romieu

**Affiliations:** 1 Instituto Nacional de Salud Pública, Mexico; 2 Comision Federal para la Proteccion contra Riesgos Sanitarios, SSA, Mexico; 3 Hospital Infantil de Mexico Federico Gomez, Mexico

## Abstract

**Background:**

Taffic-related air pollution has been related to adverse respiratory outcomes; however, there is still uncertainty concerning the type of vehicle emission causing most deleterious effects.

**Methods:**

A panel study was conducted among 147 asthmatic and 50 healthy children, who were followed up for an average of 22 weeks. Incidence density of coughing, wheezing and breathing difficulty was assessed by referring to daily records of symptoms and child's medication. The association between exposure to pollutants and occurrence of symptoms was evaluated using mixed-effect models with binary response and poisson regression.

**Results:**

Wheezing was found to relate significantly to air pollutants: an increase of 17.4 μg/m^3 ^(IQR) of PM_2.5 _(24-h average) was associated with an 8.8% increase (95% CI: 2.4% to 15.5%); an increase of 34 ppb (IQR) of NO_2 _(1-h maximum) was associated with an 9.1% increase (95% CI: 2.3% to16.4%) and an increase of 48 ppb (IQR) in O_3 _levels (1 hr maximum) to an increase of 10% (95% CI: 3.2% to 17.3%). Diesel-fueled motor vehicles were significantly associated with wheezing and bronchodilator use (IRR = 1.29; 95% CI: 1.03 to 1.62, and IRR = 1.32; 95% CI: 0.99 to 1.77, respectively, for an increase of 130 vehicles hourly, above the 24-hour average).

**Conclusion:**

Respiratory symptoms in asthmatic children were significantly associated with exposure to traffic exhaust, especially from natural gas and diesel-fueled vehicles.

## Background

In Mexico City as well as in other cities, several studies have documented the adverse effect on respiratory health, caused by exposure to air pollutants [1]. This effect appears to be greater among asthmatic children, among whom increase in respiratory symptoms, acute decrease in lung function and and an increase in emergency visits have been reported [2-4]. In Mexico City, asthma exacerbation represents 7.8% of emergency room visits at the Hospital Infantil de Mexico "Federico Gomez", the largest children's hospital in Mexico City [5]. In 2003, the Ministry of Health reported that asthma was the 12th cause of death among children under 5 years of age, and the 20th cause of death among children between 5 and 14 years old [6].

Experimental evidence in animals and humans has suggested that diesel may have a greater negative impact on respiratory health than other air pollutants [7]. The Mexico City Metropolitan area is one of the biggest and most polluted urban areas in the world and close to 85% of air pollutants in this area come from motor vehicles. One of the main pollutants consists of fine particles (PM_2.5_), 20% of which comes from industry, 32% from diesel and 25% from gasoline [8]. In vitro experimental studies have shown the toxic effects of particles collected in Mexico City [9,10]. However, no specific data exists indicating the impact of traffic exhaust, particularly diesel on the respiratory health of asthmatic and healthy children, residing in Mexico City. Therefore, we carried out a prospective longitudinal study (EVA, Emission vehicular and asthma study) in order to evaluate the effect of exposure to traffic-related air pollutants, such as PM_2.5_, nitrogen dioxide (NO_2_) and elemental carbon. These factors were related to respiratory symptoms and medication use among asthmatic and healthy children, resident in the southeastern area of Mexico City, where there is heavy truck traffic.

## Methods

### Study population

The design of this study has already been described [11]. In brief, one hundred and fity eight asthmatic children, attending the Hospital Infantil de Mexico Federico Gómez, one of the largest pediatric hospitals in the city (Mexico City) were invited to participate in the study. The diagnosis and severity of their asthma was assessed in terms of clinical symptoms and response to treatment and rated by a pediatric allergist as either mild (intermittent or persistent), moderate, or severe, following the guidelines described in the Global Initiative for Asthma (GINA) (Global Initiative for Asthma 2006) [12]. Fifty nonasthmatic children were recruited on a voluntary basis, by requesting that each asthmatic child should invite a schoolmate or a friend from their neighborhood. The children in each group ranged between 6 and 14 years of age. They lived in the study area, attended public schools located close to their homes, their attendance was voluntary and they were not selected using probability-based sampling.

Asthmatic and healthy children were recruited between July 2003 and March 2005 and followed up for an average of 22 weeks and evaluated at the same hospital every 2 weeks. At the beginning of the study, parents completed a general-purpose questionnaire, (adapted from existing survey material), outlining sociodemographic variables, past health history, and potential indoor environmental exposures (tobacco smoke and pets in the home). Information concerning allergy test results, medication, and medical visits during the 2 preceding years was obtained from the medical record. At the first visit and every 15 days thereafter, children were given a symptoms diary to be filled out by the mother. This diary was reviewed by the health staff at each visit. All procedures were explained to the parents, who signed an informed consent form. The children also gave their informed assent. Complete data from the daily dairies was available for the147 asthmatic children and 50 healthy children and this was included in the analysis.

### Respiratory symptoms

Daily records from the health diary provided information on the presence or absence of coughing, wheezing (defined as wheezing and/or difficulty breathing) and on the child's medication use (corticosteroids, such as beclometasone and flixotide among others, and bronchodilators for example salbutamol, as well as others).

### Exposure assessment

Exposure was estimated from outdoor PM_2.5 _(particulate matter < 2.5 μm in aerodynamic diameter), NO_2 _and O_3 _concentrations, as recorded by the Mexico City government at four fixed-sites for central monitoring [Red Automática de Monitoreo Afmosférico (RAMA)] at locations within the study area (Cerro de la Estrella and Hangares, Merced, Universidad Autonoma Metropolitana, Iztapalapa and La Perla). Daily average, maximum moving average and 8-hr maximum ozone, nitrogen dioxide, and PM_2.5 _concentrations along with meteorologic data (temperature and humidity) were obtained for all (505) days, during the study period. Sulfur dioxide and carbon monoxide were not taken into consideration because of the low levels of these pollutants during the study period and their high correlation with other pollutants (correlation CO with NO_2 _r = 0.65 p < 0.00 and correlation of SO_2 _and PM_2.5 _r = 0.47 p < 0.00).

The home of each participating child was georeferenced using a geographic information system (GIS), and each child was assigned to the closest monitoring station. All children attended public schools, located close to their homes, and no fixed-site monitoring station was located > 5 km from a child's residence or school.

We also conducted monitoring at each school for three 15-day periods during the follow-up, in order to validate data obtained from the fixed-site monitoring stations (RAMA). Local, daily 24-hr average PM_2.5 _was determined using Mini-Vol portable air samplers (version 4.2; Airmetrics, Eugene, Oregon, USA) with 47-mm Teflon filters (R2PJ047; Pall Gelman, Ann Arbor, MI, USA) and flows set at 5 L/min, and 7-day integrated data for NO_2 _and O_3 _concentrations were obtained using Ogawa passive samplers (Ogawa USA, Pompano Beach, FL, USA) [13]. Samplers were located outside the 37 schools, usually on the roof, at a height of up to 4 m and far from any objects (e.g., trees, buildings) that might prevent air flow. Gravimetric analysis of the 47-mm Teflon filtres was performed at the air laboratory of the National Center for Environmental Research and Training (CENICA), in Mexico City. The NO_2 _and O_3 _filters were assembled at the Mexico City laboratory. Following exposure, all badges were placed in sealed bags and sent to the Harvard School of Public Health for chemical analysis [14]. In addition, in a subsample of filters (n = 207, 11.5%), PM_2.5 _absorbance (as a marker of diesel "soot") was measured according to the procedures previously described by Watson,[13]. Black or elemental carbon (EC) mostly originates from incomplete combustion of fossil fuels and is the main factor for light particle absorption. The absorbtion coefficient is a good marker for EC when compared with the variability of PM_2.5 _levels and absorbance of PM_2.5 _filters provide reliable information on the variability of EC levels related to traffic exhaust [15]. By referring to a digital map of the Iztapalapa area (ArcView software), we selected the intersections between avenues, close to the children's residences and carried out traffic counts, applying categories to types of motor vehicle (Table 1). Traffic density was measured with pneumatic sensors every day for one week, at 7 geographical points from May to July, 2004. The week selected was considered representative concerning traffic in that area. The average density of motor vehicles according to time of day, type of vehicle, and day of the week was calculated for the study period. Vehicle type was defined, depending on the type of fuel used, as follows: A: Private cars (Gasoline); B: Small buses for public transportation (SBPT) (Gasoline or natural gas); C: School buses, other buses, pick up trucks and heavy trucks (Diesel) because our intention was to evaluate whether diesel-fueled vehicles had a greater impact on respiratory symptoms, than vehicles using other types of fuel.

**Table 1:** Air pollutants, climatic variables and traffic density during the study period

	Mean (SD)	Interquartile Range
1 hr maximum, O_3 _(ppb)	86.5(34.4)	48.0
1 hr maximum, NO_2_(ppb)	68.6(25.8)	34.0
24-hr average, PM_2.5 _(μg/m^3^)	27.8(14.9)	17.5
PM_2.5 _absorbance (10 ^-5^m^-1^)^&^	10.3(4.9)	8.5
Temperature minimum, (°C)	11.3(3.1)	4.2
Humidity minimum	34.8(11.6)	18.1
Traffic density, hourly average^†^		
A	423.0(303.1, 1840.2)	1537.1
B	30.1(13.1, 512.5)	499.5
C	20.1(14.2, 144.5)	130.3
Distance from the main avenue to the child's residence(m) ^†^	147.0(67.9, 279.9)	212.0^♣^

^† ^Median (Q25, Q75). Definition: Standard Deviation (SD). A: Private cars (Gasoline); B: Small buses for public transportation (Gasoline or natural gas); C: School buses, other buses, pick up trucks and heavy trucks (Diesel); Ozone(O3), Nitrogen dioxide(NO2) and  particles less than 2.5 μg/m^3 ^in diameter(PM_2.5_),

^&^Light absorption in inverse meters.

^♣ ^95% of the children lived less than 500 m from the main avenue

The protocol for this study was approved by the biosecurity, ethics and research committees of the participating institutions (Instituto Nacional de Salud Publica de Mexico and Hospital Infantil de Mexico "Federico Gomez", Hospital Pediatrico Iztapalapa).

### Statistical analysis

A bivariate analysis was carried out, where the basal characteristics of asthmatic and healthy children were compared, using the t-test (under the normality assumption), the Fisher exact test, or the χ^2 ^test where appropriate. The incidence density of symptoms and bronchodilator use episodes were calculated with reference to the health diary. An epidsode was defined as a manifestation of respiratory symptoms or the need for bronchidilator use, during 2 or more days and a child had to be free of symptoms or bronchodilator use for at least 3 days in order for the initiation of a new episode to be defined.

In order to analyze the occurrence of daily symptoms or bronchodilator use, we employed mixed models with random intercept, with a binary response for longitudinal data, in order to determine the effects of a pollutant (O_3_, NO_2_, PM_2.5_) [16]. The models were run, evaluating pollution effects on the same day and as a result of days prior to the occurrence of the event, assessing accumulation lags for pollutants (from 2 to 5 days). Models were adjusted for sex, severity of asthma, atopy, minimum temperature from the previous day and chronologic time. Other variables such as age, body mass index (weight/height^2^), socioeconomic status, outdoor activities, exposure to environmental tobacco smoke, pets, carpet in the home, and season did not modify the regression coefficients by more than 1%. The goodness of fit for longitudinal models was evaluated and χ^2 ^values relative to deviance in each of these models did not indicate any lack of adjustment [16]. We also determined the association between respiratory symptoms and the amount of PM_2.5 _absorbed in a subsample of filters collected in 20 schools. The analysis included 66 children for a total of 376 days of follow up (mean of 6 days per child), using mixed models.

Poisson models were used in order to analyze the association of road traffic with the incidence of symptoms and bronchodilatador use [17]. The dependent variable included the number of new events of respiratory symptoms or bronchodilator use. The exposure variable included the assigned vehicle count, considering the nearest avenue to the child's residence (that is because traffic density was measured on a specific day during the study period, for each intersection selected). The time that each child participated in the study was considerated as offset. These models were also adjusted by sex, severity of asthma, atopy and the distance between the traffic road and the child's residence. We explored the absence of overdispersion and the residuals. We used STATA 9.0 (StataCorp, Texas USA 2005) for statistical analysis.

## Results

Table 2 presents the characteristics of the study population. Sixty one % of the asthmatics were male, compared to only 43.4% of the healthy children. The presence of pets and carpets at home was significantly lower among asthmatic children. The frequency of respiratory symptoms within the 12 months prior to the beginning of the study was greater among asthmatic children, of whom 56.5% were diagnosed with mild intermittent asthma, 19.1% with moderate persistent asthma and 23.8% with mild persistent asthma.

**Table 2:** Basic characteristics of the study population

Characteristics	Asthmatics (n = 147)	Nonasthmatics (n = 50)	p value
Sex (% male)	60.5	43.4	0.031
Age [years(mean ± SD)]	9.6 ± 2.1	9.3 ± 2.2	0.975
Weight (kg)^§^	38.0 (28.0, 48.0)	32.3 (26.0, 44.6)	0.064
Height (cm)^§^	139.7 (125.0, 148.0)	132.5 (126.8, 146.2)	0.343
Maternal schooling [years(mean ± SD)]	9.7 ± 3.0	9.3 ± 3.1	0.436
Paternal smoking at home (%)	53.9	47.5	0.539
Maternal smoking at home (%)	41.2	26.2	0.135
Pets at home (%)	55.8	71.7	0.043
Carpet at home (%)	15.6	34.0	0.005
Humidity at home (%)	42.2	39.2	0.711
Prick test positivity (%)	89.2	79.1	0.089
Moderate persistent asthma (%)	19.1		
Mild persistent asthma (%)	23.8		
Mild intermittent asthma (%)	56.5		
Symptoms within the past 12 months (%)			
Dry coughing	66.7	24.5	0.000
Wheezing at least one time ‡	57.8	8.3	0.001
Rhinitis ever	38.2	7.7	0.000

Q, quartile.

^§^Mann-Whitney Test [median (Q25, Q75)]

^‡^Fisher Exact Test

### Exposure to traffic

The 24-hr average PM_2.5 _level was 27.8 μg/m^3 ^(standard deviation (SD) = 14.9). The 1-hr maximum average O_3 _level was 86.5 ppb (SD = 34.4), and the 1-hr maximum average NO_2 _level was 68.6 ppb (SD = 25.8). The mean daily PM_2.5 _absorbance was 10.3 (SD = 4.9) 10^-5 ^m^-1 ^(Table 1). PM_2.5 _were significantly correlated with O_3 _and NO_2 _(r = 0.54, and 0.62, respectively). The correlation between O_3 _and NO_2 _was 0.48. Traffic density was regrouped according to vehicle type and expressed as a 24-hour hourly average. The largest fraction of vehicles consisted of private cars (76.1%); SBPT represented 15.3%, school buses and other buses, pick up trucks and heavy trucks represented 8.7% of the hourly 24-h average(data not shown).

The incidence of symptoms episode among asthmatic children during follow-up was remarkably greater than that among healthy children. The incidence of coughing episode was 3 times greater (IRR = 3.1; 95% CI: 2.8 to 3.4) among asthmatic children than healthy children, and the incidence of wheezing was 8 times greater (IRR = 8.5; 95% CI: 6.4 to 11.4) (Table 3).

**Table 3:** Incidence density of respiratory symptom episodes and medication use among children 2003–2005

	Asthmatic children			Healthy children			Incidence Ratio	
			
Symptom	events	days	ID*	events	days	ID*	IRR	95% CI
Coughing	3692	22705	16.3	400	7561	5.3	3.1	(2.8, 3.4)
Wheezing	1329	22698	5.9	52	7552	0.7	8.5	(6.4, 11.4)
Use of corticosteroids	2902	22699	12.8					
Use of bronchodilators	2638	22643	11.7					

Definition: Incidence density (ID); Incidence density ratio (IRR); Confidence Interval (CI).

* ID (per 100 children).

**Figure 1:**
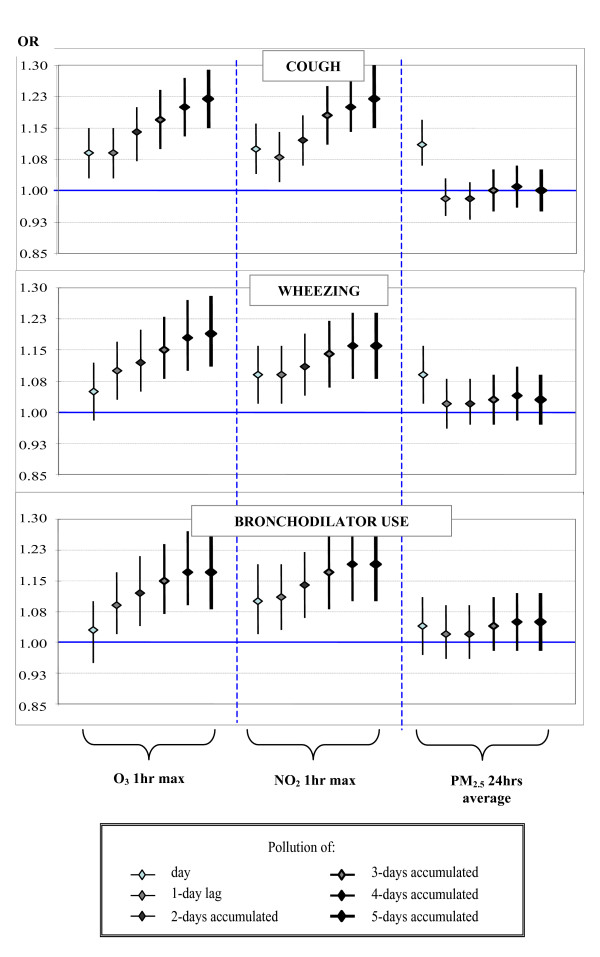
**Effect of air pollutants on respiratory symptoms and bronchodilator use among children, considering lags and cumulative exposure over several days (single pollutant models)**. OR's were calculated by using mixed models with random intercept for logistic regression, adjusting for sex, severity of asthma, atopy, minimum temperature during the previous day, and chronologic time. Symptom changes are shown with an increase of an interquartile range for each pollutant (1-hr O_3 _and 1-hr NO_2 _maximums, and 24-hr average for PM_2.5_) over several averages. * These models took into account records for symptoms at night, as well as pollution for the same day. Other models considered daily symptoms.

Among asthmatic children, coughing and wheezing at any time of the day were associated with O_3 _and NO_2 _and the effect appeared to increase with cumulative exposure over several days. For PM_2.5 _the greatest effect was observed on the same day as the exposure. The risk of coughing among asthmatics increased by 8.9% (95% CI: 3.0% to 15.1%) and the risk of wheezing increased by 10.0% (95% CI: 3.2% to 17.3%), where there was an increase of 48 ppb interquartile range (IQR) in O_3 _levels (1 hr maximum) on the previous day. These effects were stronger when cumulative exposure during 2 to 5 days was considered. The same pattern was observed for NO_2_. The risk of coughing increased by 7.5% (95% CI: 1.8% to 13.6%) and the risk of wheezing increased by 9.1% (95% CI: 2.3% to 16.4%) when NO_2 _levels (1-hr maximum) increased by 34 ppb (IQR) on the previous day, and these effects were stronger when the cumulative exposure from 2 to 5 previous days was considered (Figure 1). PM_2.5 _24-hr average levels were only significantly related to respiratory symptoms at night, when recorded that same day. An increase in the IQR (17.4 μg/m^3^) of PM_2.5 _was associated with an 11.1% increase in coughing (95% CI: 5.6% to 16.9%) and with an 8.8% increase in wheezing (95% CI: 2.4% to 15.5%). The effects of all pollutants on bronchodilator use were similar to those observed concerning symptoms (Figure 1). No significant interaction between PM_2.5_, NO_2 _or ozone and season (warm) (May to September) or cold (October to April)) was observed to effect the risk of respiratory symptoms. However the effect of ozone and PM_2.5 _on the risk of wheezing was slightly greater during the warm season.

PM_2.5 _absorbance was positively related to respiratory symptoms. An increase of an IQR (8.5 absorbance 10^-5^m^-1^) was associated with a 55% increase in coughing risk (OR = 1.55; 95% CI: 0.89 to 2.69) and with a 50% increase in wheezing risk (OR = 1.50; 95% CI: 0.83 to 2.72). Nevertheless, these associations were not significant, probably because of the small sample size employed for this subanalysis.

**Figure 2:**
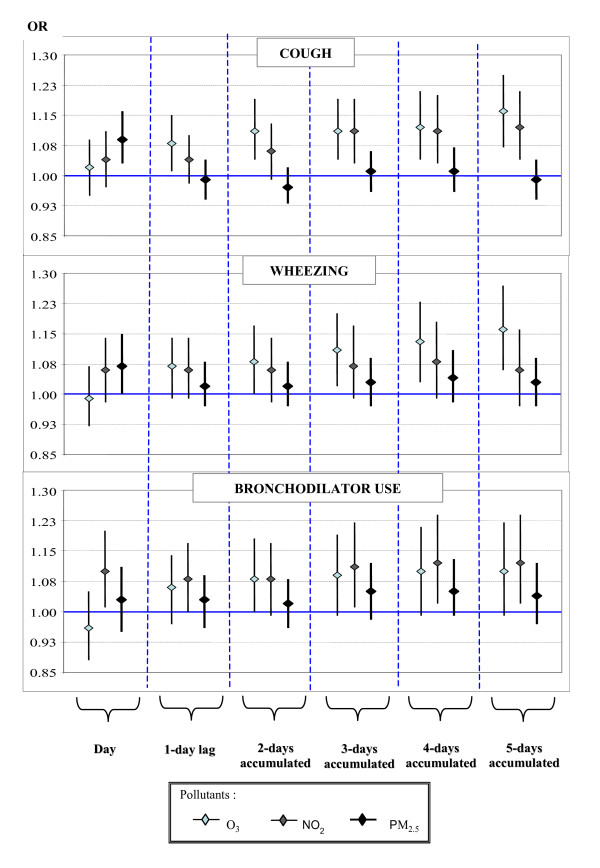
**Effect of air pollutants on respiratory symptoms and bronchodilator use among children, considering lags and cumulative exposure over several days (multi-pollutant models)**. OR's were calculated using mixed models with random intercept for logistic regression, adjusting for sex, severity of asthma, atopy, minimum temperature during the previous day, and chronologic time, and including O_3_, NO_2 _and PM_2.5 _simultaneously in the regression models. Symptom changes are shown with an increase of an interquartile range for each pollutant (1-hr O_3 _and 1-hr NO_2 _maximums, and 24-hr average for PM_2.5_) over several averages. * These models considered record of symptoms at night, and pollution for the same day. The other models considered daily symptoms.

In multipollutant models, including O_3_, NO_2 _and PM_2.5_, a similar pattern was observed. PM_2.5 _levels were significantly related to coughing on the same day as exposure. Ozone levels were significantly associated with coughing and wheezing, following a 1-day lag and these effects increased according to the number of days of cumulative exposure (2 to 5 days). NO_2 _levels were mostly related to coughing, with an increase of 11.3% (95% CI: 3.3% to 19.8%) where there was an increase of 24.5 ppb in 1-h maximum NO_2 _level, over 4 days (Figure 2).

Among healthy children, we only observed a significant association between NO_2 _levels and coughing (OR = 1. 22; 95% CI: 1.03 to 1.45 for an increase of 28.5 ppb in 1-hr maximum NO_2 _levels over 2 days). No significant association was observed between PM_2.5 _or O_3 _levels and respiratory symptoms (data not shown).

Traffic density was significantly related to respiratory symptoms (coughing and/or wheezing) in asthmatic children (Incidence rate ratio (IRR) = 1.88; 95% CI: 1.14 to 3.09 for an IQR of 499 vehicles of SBPT) (data not shown). When considering the type of vehicle, wheezing, and bronchodilator use were positively associated with the 24-hr hour average traffic count. Table 4 presents changes in the risk of each respiratory symptom, related to the type of vehicle for an interquartile (IQR) increase. Both SBPT (type B) and heavier vehicles (type C) were associated with an increase in wheezing and bronchilator use. SBPT had the greatest effect on bronchodilator use (IRR = 1.99; 95% CI: 1.10 to 3.61 for an increase of 499 SBPT (IQR)).

**Table 4:** Effect of traffic on the risk of respiratory symptoms and bronchodilator use among asthmatic children

Type of vehicle*		Coughing	Wheezing	Use of Bronchodilator
				
	IQR	IRR 95% CI+	IRR 95% CI	IRR 95% CI
A	1,537	1.17(0.87, 1.58)	1.46(0.99, 2.14)	1.64(1.01, 2.66)
B	499	1.37(0.95, 1.97)	1.80(1.12, 2.88)	1.99(1.10, 3.61)
C	130	1.15(0.97, 1.36)	1.29(1.03, 1.62)	1.32(0.99, 1.77)
Distance from main avenue to the child's residence (meters)	212	0.87(0.68, 1.11)	0.69(0.49, 0.98)	0.68(0.42, 1.08)

* Hourly average. Definition: A: Private cars (Gasoline); B: Small buses for public transportation (Gasoline or natural gas); C: School buses, other buses, pick up trucks and heavy trucks (Diesel). Poisson models are adjusted by sex, severity of asthma, atopy and the distance between the traffic road and the child's residence (this last variable is not considered in the distance model).

+ Coefficients were calculated by a change of an interquartile range (IQR).

When we considered distance to the main road as representing an index for exposure to traffic, the risk of wheezing decreased significantly with greater distance (IRR = 0.69; 95% CI: 0.49 to 0.98, with an increase in distance of 212 m (IQR)).

## Discussion

In this study, we observed that asthmatic children living in urban areas with high traffic density had a greater daily incidence of both respiratory symptoms and bronchodilator use.

The effects were present for PM_2.5_, NO_2 _and O_3 _and increased with exposure over several days in the case of NO_2 _and O_3_. Traffic count on the nearest main avenue to the child's residence was also related to respiratory symptoms.

Our results are consistent with those of previous studies, reporting on the increased risk of coughing, wheezing, breathing difficulty, medication and hospital admissions among asthmatic children, which is associated with exposure to O_3 _[18], NO_2_[19,20], particulate matter (PM_10 _and PM_2.5_)[21], and heavy truck traffic [22-24] Our study, as in the one carried out by Mar and co-workers [21], using a panel of asthmatic children, found an association between respiratory symptoms (coughing and wheezing) and particulate matter. Likewise, a prospective study carried out in southern New England [18] involving 271 asthmatic children showed that O_3 _and PM_2.5 _concentrations below those recommended by the EPA were associated with an increase in the likelihood of wheezing, chest tightness and persistent coughing among children permanently on medication. In the case of co-pollutant models, only the O_3 _effect continued to be significant, concerning symptoms and medication use. In our study, O_3 _levels were related to both coughing and wheezing and this effect persisted in the co-pollutant models. Nitrogen dioxide and PM_2.5 _are two of the pollutants most often considered in order to assess the impact of road traffic on air pollution [23,25-27] Both pollutants have been related to an increase in respiratory symptoms [28-30]. In our study, NO_2 _levels had a greater effect than PM_2.5 _and this effect increased with cumulative exposure, whereas the effect of the PM_2.5 _level was only evident on the same day as the exposure occurred. Sulfur dioxide has been related to respiratory emergency room admission among children [31]; however this effect was not manifest in multipollutant models. In our study, SO_2 _levels were low (mean 0.011 ppm, SD = 0.009) and when this pollutant was included in our models, results remained similar.

Other indicators of exposure to road traffic such as distance to main avenues and traffic density [22-24] have been shown to relate to respiratory symptoms. In our study, we found a significant increase in wheezing and use of bronchodilators, relating to an increase in traffic count. This effect was observed for all types of vehicles but was greater in the case of SBPT and buses and trucks, although separating effects according to type of vehicle may pose a problem given the correlation which exists between traffic classes (coefficient of correlations from 0.5 to 0.7). In Mexico, out of 2 million 654 thousand vehicles, 3% correspond to heavy trucks and 1% to SBPT [32] and both of these are known to be a source of PM_2.5 _and NO_2_. Many public transportation buses use natural compressed gas, which produces a greater quantity of NO_2 _than common natural gas. When accounting for the distance from the child's residence to the main avenue, both the traffic count (positively) and the distance (inversely) were significantly related to respiratory symptoms. We measured PM_2.5 _absorbance in a subsample and found a positive association between respiratory symptoms and PM_2.5 _absorbance. Although the sample size for this analysis was small, our results further support the importance of diesel exposure, as observed in other studies [30,33].

A limitation to our study lies in the fact that environmental exposure was evaluated in terms of the daily reports from the RAMA central monitoring locations, potentially causing misclassification. Although exposure to pollutants was evaluated ecologically, exposure for each child was assigned according to a spatial GIS, in order to determine the monitoring site closest to the child's residence. As children mostly lived close to their schools, we believe that our exposure assessment is valid. Additionally, we conducted local monitoring of PM_2.5 _during 15 days in each of the 37 schools. The correlation between the RAMA monitors and the school monitors was 0.77. In any event, the measurement error in exposure assessment is likely to be random and would therefore tend to underestimate the association. Participants in this study were selected on a voluntary basis and were not therefore representative of the asthmatic and healthy population of Mexican children. In fact in our sample, 60.5% of the asthmatic children were boys, whereas data from the Hospital Infantil de Mexico "Federico Gomez" suggest a ratio of 1.6 for boys versus girls, in the age range of our children (personal communication Dr. Blanca Del Rio). One advantage of our study consists in its prospective nature, as this permitted an assessment of the association between daily changes in air pollution levels and symptoms, by using longitudinal data analysis techniques, including random effects to represent specific differences in children's health responses (symptoms), over time. Our results remained similar after adjusting for home characteristics, such as the presence of pets or carpeting in the children's room and climatic variables. The consistency of our results regarding air pollutant levels and traffic count, in relation to respiratory symptoms and bronchodilator use indicates a causal relationship.

The biological factors that may account for the occurrence of respiratory symptoms associated with exposure to air pollutants are not completely understood. Some studies suggest that an increase in the inflammatory response could account for this association [34,35], particularly among susceptible individuals, such as asthmatic children. Oxidative stress has been shown to be a major underlying feature of the toxic effect of air pollutants. It acts as a trigger for a number of redox sensitive signaling pathways, such as inflammatory response and citokine production [36-39]. Toxicity may therefore be caused by an imbalance of biologic pro-oxidants and antioxidant processes [40] linked to increased exposure to oxidants or to the presence of impaired antioxidant defenses. In addition, some genetic characteristics may also increase susceptibility to air pollutants [41]. However, better understanding of the physiological effects of exposure to air pollutants, particularly diesel, is required in order to improve the prevention and control of respiratory impairment related to exposure, especially among asthmatic children.

## Conclusion

Our prospective data indicate that among asthmatic children, traffic-related air pollutants correlate with an increase in coughing, wheezing and bronchodilator use. Among healthy children an increase in coughing was observed. During the study period, the Mexican norm[42] for PM_2.5 _and ozone were exceeded on 7.2% and 43.3% of the days, respectively, whereas PM_2.5 _was exceeded on 48.1% of days, when the EPA standard was referred to [42]. Given the important adverse effects of these traffic exhaust pollutants, greater, stringent control of vehicular emission and traffic is required, especially close to schools and in areas where children participate in outdoor activities. These results have significant public health policy implications, as a large proportion of schools in Mexico City and other countries are located very close to roads carrying heavy traffic.

## Competing interests

The authors declare that they have no competing interests.

## Authors' contributions

CE performed all analyses and wrote the initial draft of the paper. IR obtained funding for the project, conceived the premise and directed writing and analysis.

AB, LH, HM, MR, JS, MC, JT and BR participated in funding, data collection, data analysis and interpretation, and editing.

All authors have read and approved the final manuscript.
